# Supplementary figures and images for: Myosin 1f and Proline-rich 13 are transcriptionally upregulated yet functionally redundant in CD4+ T cells during blood-stage Plasmodium infection
Source: PLoS One. 2025 Mar 25;20(3):e0320375. doi: 10.1371/journal.pone.0320375 (PMC11936294; doi:10.1371/journal.pone.0320375)

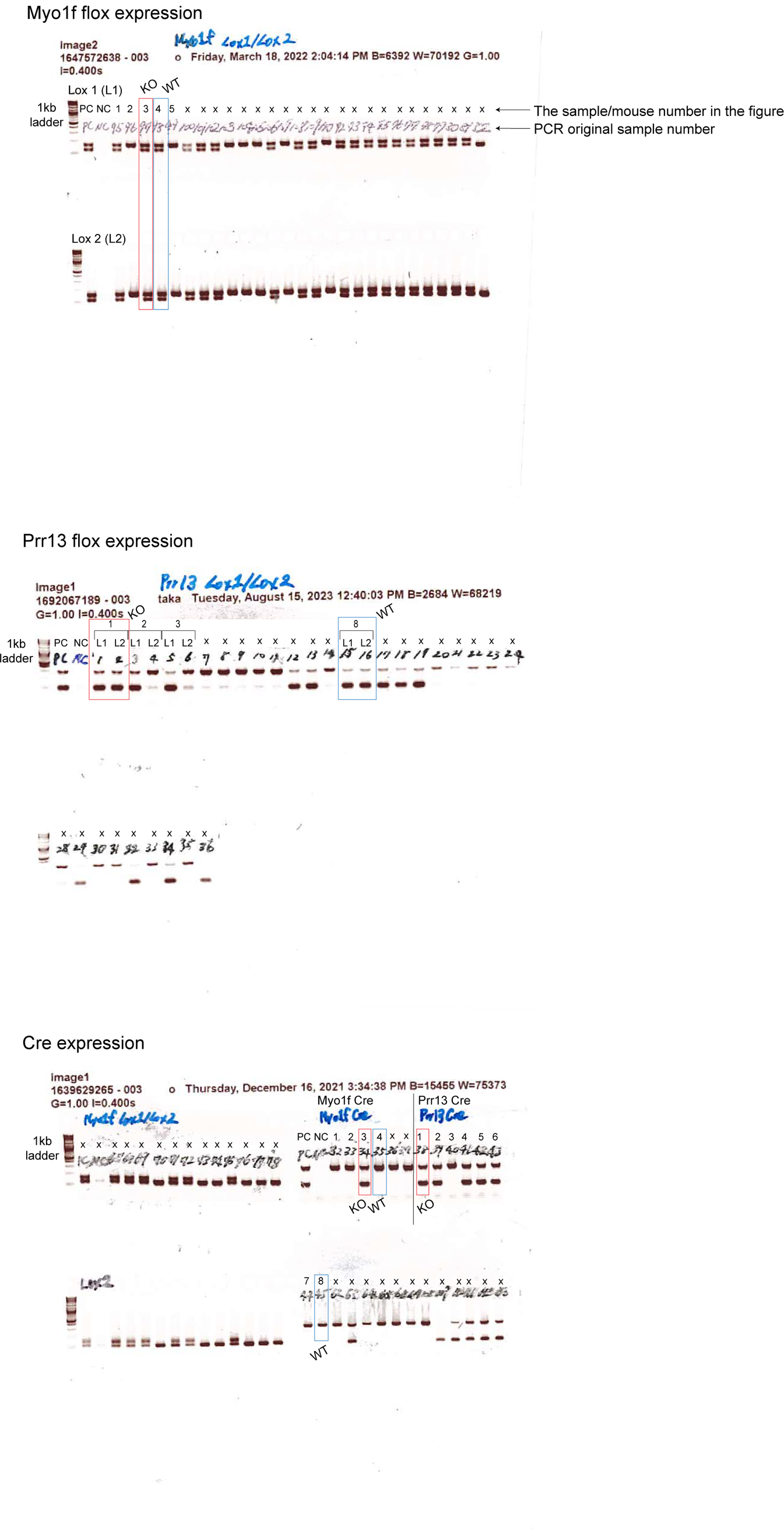

Supplement: S1 Fig — (TIF) [file pone.0320375.s001.tif]
