# Supplementary material for: Myosin 1f and Proline-rich 13 are transcriptionally upregulated yet functionally redundant in CD4+ T cells during blood-stage Plasmodium infection
Source: PLoS One. 2025 Mar 25;20(3):e0320375. doi: 10.1371/journal.pone.0320375 (PMC11936294; doi:10.1371/journal.pone.0320375)
Supplement: Attachment — Submitted filename: Response to Reviewers_Rebuttal Letter.docx [file pone.0320375.s003.docx]

Rebuttal Letter

PLOS ONE PONE-D-24-38309

Myosin 1f and Proline-rich 13 are transcriptionally upregulated yet functionally redundant in T helper cells during experimental malaria.

Editor’s comments

Dear Dr. Haque,

Your manuscript was reviewed by two experts in the field and both of them were enthusiastic about the findings reported in your manuscript. There were, however, some concerns that were raised by one of the reviewers that need to be addressed prior to making a final decision on your manuscript. As such, my recommendation is minor revision. Therefore, we invite you to submit a revised version of the manuscript that addresses the points raised during the review process.

Please submit your revised manuscript by Dec 07 2024 11:59PM. If you will need more time than this to complete your revisions, please reply to this message or contact the journal office at [plosone@plos.org](mailto:plosone@plos.org). When you're ready to submit your revision, log on to <https://www.editorialmanager.com/pone/> and select the 'Submissions Needing Revision' folder to locate your manuscript file.

Please include the following items when submitting your revised manuscript:

- A rebuttal letter that responds to each point raised by the academic editor and reviewer(s). You should upload this letter as a separate file labeled 'Response to Reviewers'.
- A marked-up copy of your manuscript that highlights changes made to the original version. You should upload this as a separate file labeled 'Revised Manuscript with Track Changes'.
- An unmarked version of your revised paper without tracked changes. You should upload this as a separate file labeled 'Manuscript'.

If you would like to make changes to your financial disclosure, please include your updated statement in your cover letter. Guidelines for resubmitting your figure files are available below the reviewer comments at the end of this letter.

If applicable, we recommend that you deposit your laboratory protocols in [protocols.io](http://protocols.io/) to enhance the reproducibility of your results. Protocols.io assigns your protocol its own identifier (DOI) so that it can be cited independently in the future. For instructions see: [https://journals.plos.org/plosone/s/submission-guidelines#loc-laboratory-protocols](https://track.editorialmanager.com/CL0/https:%2F%2Fjournals.plos.org%2Fplosone%2Fs%2Fsubmission-guidelines%23loc-laboratory-protocols/1/010f0192ba66049c-59436351-2d41-492d-8b71-b71129ab26b0-000000/CtzpRhBb8MT-Q-dfgJgEG8lomHbt_nVJtRXj1LbtAyE=182). Additionally, PLOS ONE offers an option for publishing peer-reviewed Lab Protocol articles, which describe protocols hosted on [protocols.io](http://protocols.io/). Read more information on sharing protocols at [https://plos.org/protocols?utm_medium=editorial-email&utm_source=authorletters&utm_campaign=protocols](https://track.editorialmanager.com/CL0/https:%2F%2Fplos.org%2Fprotocols%3Futm_medium=editorial-email%26utm_source=authorletters%26utm_campaign=protocols/1/010f0192ba66049c-59436351-2d41-492d-8b71-b71129ab26b0-000000/9kzTSVrSqmO5ZXU9FoFNa5Wk8_KFOguv0gelF25yeVs=182).

We look forward to receiving your revised manuscript.

Kind regards,

Dr. Joseph J Mattapallil
Academic Editor
PLOS ONE

We thank the editor and reviewers for their comments and suggestions on our manuscript. We have thoroughly addressed all concerns. Below, we provide a point-by-point response to the reviewers’ comments, with our responses highlighted in BLUE text. Where appropriate, we have included new text that have been incorporated into the revised manuscript, along with line numbers for ease of reference.

Journal Requirements:

[When submitting your revision, we need you to address these additional requirements.

1. Please ensure that your manuscript meets PLOS ONE's style requirements, including those for file naming. The PLOS ONE style templates can be found at https://journals.plos.org/plosone/s/file?id=wjVg/PLOSOne_formatting_sample_main_body.pdf and](http://track.editorialmanager.com/CL0/file:%2F%2F%2FC:%2FUsers%2Fe754871%2FDesktop%2FPRTC%2FPRTC%25202024%2FOctober%25202024%2FOctober%252023,%25202024%2FPONE-D-24-38309%2FJournal%2520requirements:%2520%250b%250bWhen%2520submitting%2520your%2520revision,%2520we%2520need%2520you%2520to%2520address%2520these%2520additional%2520requirements.%2520%250b%250b1.%2520Please%2520ensure%2520that%2520your%2520manuscript%2520meets%2520PLOS%2520ONE's%2520style%2520requirements,%2520including%2520those%2520for%2520file%2520naming.%2520The%2520PLOS%2520ONE%2520style%2520templates%2520can%2520be%2520found%2520at%2520https:%2Fjournals.plos.org%2Fplosone%2Fs%2Ffile%253fid=wjVg%2FPLOSOne_formatting_sample_main_body.pdf%2520and/1/010f0192ba66049c-59436351-2d41-492d-8b71-b71129ab26b0-000000/18q7r6Zn4VmM3INTOjYICFyZMiDEsNA-XSN6bgPSe0s=182) [https://journals.plos.org/plosone/s/file?id=ba62/PLOSOne_formatting_sample_title_authors_affiliations.pdf](https://track.editorialmanager.com/CL0/https:%2F%2Fjournals.plos.org%2Fplosone%2Fs%2Ffile%3Fid=ba62%2FPLOSOne_formatting_sample_title_authors_affiliations.pdf/1/010f0192ba66049c-59436351-2d41-492d-8b71-b71129ab26b0-000000/SwDBkWqYyLbY-lkKhvws2syGlVZIXYlby0V3DP5QZ90=182)

We have revised the manuscript to follow PLOS ONE's style requirements

2. To comply with PLOS ONE submissions requirements, in your Methods section, please provide additional information regarding the experiments involving animals and ensure you have included details on methods of anesthesia and/or analgesia.

In the revised Methods section, we have now provided additional information regarding animal care and welfare as follows (line 378): “All animal experiments were conducted on female mice....” Specifically, we have included details on the monitoring criteria used for humane endpoints, the method of euthanasia, and the approvals obtained from the University of Melbourne Animal Ethics Committee. As anesthesia or analgesia was not required for our experimental protocol, we have specified that all procedures were conducted with minimal distress to the mice.

3. [We note that Figure 1 in your submission contain map/satellite images which may be copyrighted. All PLOS content is published under the Creative Commons Attribution License (CC BY 4.0), which means that the manuscript, images, and Supporting Information files will be freely available online, and any third party is permitted to access, download, copy, distribute, and use these materials in any way, even commercially, with proper attribution. For these reasons, we cannot publish previously copyrighted maps or satellite images created using proprietary data, such as Google software (Google Maps, Street View, and Earth). For more information, see our copyright guidelines: http://journals.plos.org/plosone/s/licenses-and-copyright. We require you to either (a) present written permission from the copyright holder to publish these figures specifically under the CC BY 4.0 license, or (b) remove the figures from your submission:

a. You may seek permission from the original copyright holder of Figure 1 to publish the content specifically under the CC BY 4.0 license. We recommend that you contact the original copyright holder with the Content Permission Form (http://journals.plos.org/plosone/s/file?id=7c09/content-permission-form.pdf) and the following text: “I request permission for the open-access journal PLOS ONE to publish XXX under the Creative Commons Attribution License (CCAL) CC BY 4.0 (http://creativecommons.org/licenses/by/4.0/). Please be aware that this license allows unrestricted use and distribution, even commercially, by third parties. Please reply and provide explicit written permission to publish XXX under a CC BY license and complete the attached form.” Please upload the completed Content Permission Form or other proof of granted permissions as an "Other" file with your submission. In the figure caption of the copyrighted figure, please include the following text: “Reprinted from [ref] under a CC BY license, with permission from [name of publisher], original copyright [original copyright year].”

b. If you are unable to obtain permission from the original copyright holder to publish these figures under the CC BY 4.0 license or if the copyright holder’s requirements are incompatible with the CC BY 4.0 license, please either i) remove the figure or ii) supply a replacement figure that complies with the CC BY 4.0 license. Please check copyright information on all replacement figures and update the figure caption with source information. If applicable, please specify in the figure caption text when a figure is similar but not identical to the original image and is therefore for illustrative purposes only. The following resources for replacing copyrighted map figures may be helpful: USGS National Map Viewer (public domain): http://viewer.nationalmap.gov/viewer/ The Gateway to Astronaut Photography of Earth (public domain): http://eol.jsc.nasa.gov/sseop/clickmap/ Maps at the CIA (public domain): https://www.cia.gov/library/publications/the-world-factbook/index.html and https://www.cia.gov/library/publications/cia-maps-publications/index.html NASA Earth Observatory (public domain): http://earthobservatory.nasa.gov/ Landsat: http://landsat.visibleearth.nasa.gov/ USGS EROS (Earth Resources Observatory and Science (EROS) Center) (public domain): http://eros.usgs.gov/# Natural Earth (public domain):](http://track.editorialmanager.com/CL0/file:%2F%2F%2FC:%2FUsers%2Fe754871%2FDesktop%2FPRTC%2FPRTC%25202024%2FOctober%25202024%2FOctober%252023,%25202024%2FPONE-D-24-38309%2FWe%2520note%2520that%2520Figure%25201%2520in%2520your%2520submission%2520contain%2520map%2Fsatellite%2520images%2520which%2520may%2520be%2520copyrighted.%2520All%2520PLOS%2520content%2520is%2520published%2520under%2520the%2520Creative%2520Commons%2520Attribution%2520License%2520(CC%2520BY%25204.0),%2520which%2520means%2520that%2520the%2520manuscript,%2520images,%2520and%2520Supporting%2520Information%2520files%2520will%2520be%2520freely%2520available%2520online,%2520and%2520any%2520third%2520party%2520is%2520permitted%2520to%2520access,%2520download,%2520copy,%2520distribute,%2520and%2520use%2520these%2520materials%2520in%2520any%2520way,%2520even%2520commercially,%2520with%2520proper%2520attribution.%2520For%2520these%2520reasons,%2520we%2520cannot%2520publish%2520previously%2520copyrighted%2520maps%2520or%2520satellite%2520images%2520created%2520using%2520proprietary%2520data,%2520such%2520as%2520Google%2520software%2520(Google%2520Maps,%2520Street%2520View,%2520and%2520Earth).%2520For%2520more%2520information,%2520see%2520our%2520copyright%2520guidelines:%2520http:%2Fjournals.plos.org%2Fplosone%2Fs%2Flicenses-and-copyright.%2520We%2520require%2520you%2520to%2520either%2520(a)%2520present%2520written%2520permission%2520from%2520the%2520copyright%2520holder%2520to%2520publish%2520these%2520figures%2520specifically%2520under%2520the%2520CC%2520BY%25204.0%2520license,%2520or%2520(b)%2520remove%2520the%2520figures%2520from%2520your%2520submission:%2520%250b%250ba.%2520You%2520may%2520seek%2520permission%2520from%2520the%2520original%2520copyright%2520holder%2520of%2520Figure%25201%2520to%2520publish%2520the%2520content%2520specifically%2520under%2520the%2520CC%2520BY%25204.0%2520license.%2520We%2520recommend%2520that%2520you%2520contact%2520the%2520original%2520copyright%2520holder%2520with%2520the%2520Content%2520Permission%2520Form%2520(http:%2Fjournals.plos.org%2Fplosone%2Fs%2Ffile%3Fid=7c09%2Fcontent-permission-form.pdf)%2520and%2520the%2520following%2520text:%2520%25E2%2580%259CI%2520request%2520permission%2520for%2520the%2520open-access%2520journal%2520PLOS%2520ONE%2520to%2520publish%2520XXX%2520under%2520the%2520Creative%2520Commons%2520Attribution%2520License%2520(CCAL)%2520CC%2520BY%25204.0%2520(http:%2F%2Fcreativecommons.org%2Flicenses%2Fby%2F4.0%2F).%2520Please%2520be%2520aware%2520that%2520this%2520license%2520allows%2520unrestricted%2520use%2520and%2520distribution,%2520even%2520commercially,%2520by%2520third%2520parties.%2520Please%2520reply%2520and%2520provide%2520explicit%2520written%2520permission%2520to%2520publish%2520XXX%2520under%2520a%2520CC%2520BY%2520license%2520and%2520complete%2520the%2520attached%2520form.%25E2%2580%259D%2520Please%2520upload%2520the%2520completed%2520Content%2520Permission%2520Form%2520or%2520other%2520proof%2520of%2520granted%2520permissions%2520as%2520an%2520%2522Other%2522%2520file%2520with%2520your%2520submission.%2520In%2520the%2520figure%2520caption%2520of%2520the%2520copyrighted%2520figure,%2520please%2520include%2520the%2520following%2520text:%2520%25E2%2580%259CReprinted%2520from%2520%255bref%255d%2520under%2520a%2520CC%2520BY%2520license,%2520with%2520permission%2520from%2520%255bname%2520of%2520publisher%255d,%2520original%2520copyright%2520%255boriginal%2520copyright%2520year%255d.%25E2%2580%259D%2520%250b%250bb.%2520If%2520you%2520are%2520unable%2520to%2520obtain%2520permission%2520f/1/010f0192ba66049c-59436351-2d41-492d-8b71-b71129ab26b0-000000/QvyBfsPRQQeOFc-ZT1Bgl_hVr6oEoGUmB9YSLfsLomY=182" \t "_blank) [http://www.naturalearthdata.com/](http://track.editorialmanager.com/CL0/http:%2F%2Fwww.naturalearthdata.com%2F/1/010f0192ba66049c-59436351-2d41-492d-8b71-b71129ab26b0-000000/jTdtGhW4zX6OvAMiVF7I3ONraiuTyMtBSxpoFssVFBg=182)

We previously consulted Dr. Nick Bernard, a Senior Editor at Nature Immunology regarding the reuse of figures from the study by Soon et al. (ref. 11) in publications with Cell Reports (ref. 38) and Nature Communications (ref. 15). In those instances, they confirmed that providing a citation was sufficient, with no additional permissions or paperwork required. The permission statements in the figure legend of Figure. 1 as follows (line 138); “(A-D) Reprinted from....”

4. Please upload a copy of Supporting Information Figure 1 which you refer to in your text on page 18.

Thank you for pointing this out, and we apologize for any confusion. This was an error in the original manuscript. The reference to "Supporting Information Figure 1" was intended to refer to Figure 2C, as we decided not to include supplementary figures. We have corrected this in the revised manuscript for clarity (line 367)

5. PLOS ONE now requires that authors provide the original uncropped and unadjusted images underlying all blot or gel results reported in a submission’s figures or Supporting Information files. This policy and the journal’s other requirements for blot/gel reporting and figure preparation are described in detail at <https://journals.plos.org/plosone/s/figures#loc-blot-and-gel-reporting-requirements> and <https://journals.plos.org/plosone/s/figures#loc-preparing-figures-from-image-files>. When you submit your revised manuscript, please ensure that your figures adhere fully to these guidelines and provide the original underlying images for all blot or gel data reported in your submission. See the following link for instructions on providing the original image data: <https://journals.plos.org/plosone/s/figures#loc-original-images-for-blots-and-gels>. In your cover letter, please note whether your blot/gel image data are in Supporting Information or posted at a public data repository, provide the repository URL if relevant, and provide specific details as to which raw blot/gel images, if any, are not available. Email us at [plosone@plos.org](mailto:plosone@plos.org) if you have any questions.

We have submitted the original, uncropped, and unadjusted gel images as the S1_raw_image file alongside the revised manuscript. In addition, the new Figure 3C in the revised version now retains the original gel results with minimal cropping and adjustment, ensuring transparency and preserving the integrity of the data.

6. Please review your reference list to ensure that it is complete and correct. If you have cited papers that have been retracted, please include the rationale for doing so in the manuscript text, or remove these references and replace them with relevant current references. Any changes to the reference list should be mentioned in the rebuttal letter that accompanies your revised manuscript. If you need to cite a retracted article, indicate the article’s retracted status in the References list and also include a citation and full reference for the retraction notice.

Thank you for the reminder. We have reviewed the reference list to ensure its accuracy.

Reviewer's Responses to Questions

1. Is the manuscript technically sound, and do the data support the conclusions?

The manuscript must describe a technically sound piece of scientific research with data that supports the conclusions. Experiments must have been conducted rigorously, with appropriate controls, replication, and sample sizes. The conclusions must be drawn appropriately based on the data presented.

Reviewer #1: Partly

Reviewer #2: Yes

2. Has the statistical analysis been performed appropriately and rigorously?

Reviewer #1: Yes

Reviewer #2: Yes

3. Have the authors made all data underlying the findings in their manuscript fully available?

The [PLOS Data policy](http://track.editorialmanager.com/CL0/http:%2F%2Fwww.plosone.org%2Fstatic%2Fpolicies.action%23sharing/1/010f0192ba66049c-59436351-2d41-492d-8b71-b71129ab26b0-000000/rhwQoO86UcOGvfauQ1SbH4VbEKcfVTNe7WqvQ6dpCo4=182) requires authors to make all data underlying the findings described in their manuscript fully available without restriction, with rare exception (please refer to the Data Availability Statement in the manuscript PDF file). The data should be provided as part of the manuscript or its supporting information, or deposited to a public repository. For example, in addition to summary statistics, the data points behind means, medians and variance measures should be available. If there are restrictions on publicly sharing data—e.g. participant privacy or use of data from a third party—those must be specified.

Reviewer #1: No

Reviewer #2: Yes

4. Is the manuscript presented in an intelligible fashion and written in standard English?

PLOS ONE does not copyedit accepted manuscripts, so the language in submitted articles must be clear, correct, and unambiguous. Any typographical or grammatical errors should be corrected at revision, so please note any specific errors here.

Reviewer #1: Yes

Reviewer #2: Yes

5. Reviewer's Comments to the Author

**Reviewer #1:**

Positive feedback. I applaud the authors for moving forward with a publication of negative results. It is important to show that some suspected genes are not important in T cell responses (to malaria). The text is well written, figures are of a good quality (but see comments), and results seems solid (given performed experiments, see also below). I also appreciated authors showing the data from individual mice; this is important and should be the standard in the field.

**Comments.**

1. The rationalization of choosing these two genes to study is still poor. Ok, these genes are unregulated in scRNAseq data. So what? I predict hundreds of genes are unregulated - why these two? Are they unregulated to the highest level (mRNA)? Are they upregulated at the protein level? I found the data presented in Fig 1A being unclear. NOTE: because individual panels are not labeled, it is hard to refer to individual panels. But, in Fig 1A trajectories - how uncertain are these? In Fig 1A violin plots -> what are these (no labels given)? No stats are given either.

Thank you for your question regarding the rationale behind our selection of Myo1f and Prr13 for this study. Our choice was driven by both the strong upregulation of these genes in our scRNA-seq dataset and functional insights from prior studies, which pointed to their potential roles in T cell biology during infection.

For Myo1f, previous studies have demonstrated its role in controlling cell motility and granule exocytosis in neutrophils and other innate immune cells (ref. 21, 24-27). This evidence led us to hypothesize that Myo1f might similarly regulate T cell movement and function during infection. Its consistent high expression specifically in effector and memory CD4^+^ T cells during blood-stage malaria suggested that Myo1f could influence T cell movement and/or function in experimental malaria, making it a compelling candidate for further investigation. We have addressed this point by revising the text as follows (line 111): “Given prior evidence of Myo1f’s role....”

For Prr13, sequence analyses (UniProt and NCBI) and prior studies (ref. 31, 33-35) indicate that it may encode a DNA-binding protein localized in the nucleus with transcriptional regulatory functions. This evidence led us to hypothesize that Prr13 might act as a transcriptional factor in T cells, potentially controlling differentiation and cellular maintenance. Thus, the distinct and elevated expression of Prr13 in effector and memory T cells pointed toward a possible role in T cell fate decisions and memory maintenance in experimental blood-stage malaria. We have addressed this point by revising the text as follows (line 118): “Given sequence predictions and prior studies suggesting that Prr13....” We hope this response clarifies our rationale and highlights the potential of Myo1f and Prr13 to provide new insights into T cell function and differentiation in the context of infection.

We apologize for the lack of labelling and clarity in Fig. 1A. In the revised figure, we have now sub-labelled each panel within Fig. 1A. The violin plots represent mRNA expression levels in PbTIIs across different time points (day 0, day 7, day 14, and day 28 post-infection) which helps identify the gene expression dynamics of *Myo1f* and *Prr13* during *Pc*AS infection. Also, we considered performing single-cell level statistics. However, this approach often results in inflated p-values, as each cell is treated as an individual sample, potentially making all comparisons appear statistically significant without reflecting biologically meaningful differences. Therefore, to provide an accurate representation of our findings, we have chosen not to perform statistical tests at the single-cell level for this data.

To address concerns about the trajectory in Fig. 1A, our previous studies (*Soon et al.*, ref. 11; *Lönnberg et al.*, ref. 14) used scRNA-seq and *in vivo* analyses to track PbTII cell differentiation during blood-stage malaria from Day 0 to Day 28. These studies established a clear timeline of CD4^+^ T cell responses, showing gene dynamics and the presence of both effector Th1/Tfh and memory T cells. Therefore, the trajectory in Fig. 1A builds on this established framework, accurately reflecting effector and mamory PbTII cell differentiation. We hope this clarifies the reliability of the trends observed in our analysis.

2. Measurements are mostly done at day 7. Why? Could it be too early to see a difference? Is there a positive control authors could present that would justify measurements at day 7 (or 28) as appropriate?

We selected Day 7 post-infection as it represents the peak of PcAS infection, a critical time when CD4+ T cells distinctly differentiate into Th1 and Tfh subsets. Previous studies by *Soon et al.* (ref. 11) and *Lönnberg et al.* (ref. 14) showed that this timepoint is marked by high expression of Th1 markers (CXCR6, T-bet, and IFN-γ) and Tfh markers (CXCR5 and Bcl-6) at both the transcriptional and protein levels. Additionally, *Soon et al.* found that IFNγ^+^ PbTII cell responses significantly decreased after Day 7 without additional stimulation, underscoring Day 7 as a critical point for assessing effector PbTII differentiation. For memory and recall responses, we chose Day 28 or Day 31 post-primary infection, when parasitemia is undetectable, offering a stable point for evaluating memory and rechallenge responses. This timing is supported by our previous work from *Soon et al.* and *Lee et al.* (ref. 15).

3. I could not understand the results shown in Fig 3D - the way I see these gels is that expression in WT vs. KO is the same, i.e., KO did not work. I think someone experienced in gels should look into these (e.g., Dr Elisabeth Bik @MicrobiomDigest on Twitter)

Thank you for raising this important point. To clarify, the difference between WT and KO groups is based on Cre expression, as both groups contain floxed alleles (f/f) for *Myo1f* or *Prr13*. In our figures, we indicate Myo1f WT as Myo1f^(f/f)^ and Myo1f KO as CD4-Cre Myo1f^(f/f)^, with a similar designation for Prr13 WT as Prr13^(f/f)^ and KO as CD4-Cre Prr13^(f/f)^.

In response to your comment, we have included the original, uncropped, and unadjusted gel images in the supplemental information for complete transparency. We also revised the manuscript to include a new Figure 3C with minimal cropping and adjustments, preserving the original gel data. This figure shows Cre expression exclusively in the KO groups, not in the WT groups, for both Myo1f and Prr13, confirming the successful establishment of WT and KO mice. Additionally, to validate the Cre/Lox system, we initially tested knockout efficacy on the gene Maf, as shown in Figure 2. This confirmed the Cre/Lox-mediated knockout system in PbTIIs in our malaria model, supporting its reliability for studying Myo1f and Prr13.

4. I am not sure about some statistical analyses showing no difference between WT and KO cells. For example, in Fig 4B and 4D (cannot point to panels!), there is a tendency of lower total T cell numbers in PbT-II KO cells. I wonder if experiments in which WT and KO cells are co-transferred to the mice and then look at the response magnitude. This way, the data will be paired and may allow for higher statistical power to detect difference.

We recognize that co-transferring WT and KO cells into the same recipient mouse would allow for a direct comparison within an identical microenvironment, helping to control for homeostatic variations and enhancing statistical power through paired analysis. However, a limitation of our current setup is that both WT and KO cells are marked as CD45.2, making it impossible to distinguish them if co-transferred into a CD45.1 (Ly5.1) recipient mouse. As a result, we conducted our experiments in separate cohorts to assess WT and KO effects independently, using unpaired, non-parametric Mann-Whitney tests for statistical analysis.

**Minor comments.**

Improvements in figures must be made. Every panel in a figure should be "named", e.g., you can use Ai, Aii, Aiii, etc to label sub-panels in a figure with multiple elements (A/B/C etc).

I highly recommend generating pdf of the text with ALL figures and figure captions being on the same page, e.g., as done in published papers or grant applications. This makes reviewing of the paper easier. Also, number the lines so reference to specific lines in the text can be made. And obvious -> have page numbers!

Thank you for pointing out these improvements. We have now labelled each figure with sub-panels (e.g., Ai, Aii, etc.) and added line numbers and page numbers to the manuscript to enhance clarity and accessibility for review.

The text in 2nd paragraph in Results is unclear. It reads as you put T cells into WT Cre-expressing mice but it does not seem to be correct as you generated KO mice first. Or perhaps I did not understand the text well -> in any case, please review and update if needed.

Thank you for highlighting this point. To clarify, our experimental setup involved generating donor PbTIIs from two types of mice: KO mice that express Cre-recombinase with the target gene flanked by loxP sites (f/f), and WT mice that only express the (f/f) alleles without Cre. These donor cells were then adoptively transferred into congenic recipient mice. We agree that the original text could be confusing and have revised it accordingly to provide a clearer explanation as follows (line 142): “In this system, donor PbTIIs were obtained either from....”

If KO T cells generate fewer Tfh cells, does this result in lower Ab (IgG?) levels to the parasites? Perhaps this needs to be shown.

Thank you for this comment. First, antigen-specific B cells that interact with Plasmodium-specific CD4+ (PbTII) Tfh cells have not yet been identified in experimental malaria, making it challenging to measure antibody titers without clearly identifiable antigen-specific B cells. Second, we observed that disrupting these genes in PbTIIs did not affect the overall polyclonal CD4+ T cell response, including Th1 and Tfh differentiation, suggesting that B cell responses and antibody production were likely unaffected by this gene disruption. However, we acknowledge that further examination of antibody responses could provide valuable insights and would consider this in future studies.

The quality of the KO is stated in Methods to be shown in Fig 2B (cannot point more precisely because page and line numbers are missing). I cannot see this. Please make sure you refer to the right figures/panels.

Thank you for pointing this out, and we apologize for the confusion. The correct reference for the cMaf KO validation is Figure 2D, not Figure 2B as previously stated. We have updated the Methods section to accurately refer to Figure 2D (line 367).

Purity of CD4 T cells was exceeding approximately 80%. What does this mean? 81%? Or 99%. Please put specific numbers/range, e.g., 80-85%.

The purity of CD4^+^ T cells was consistently between 85-90%, and we have now included this specific range in the revised manuscript as follows (line 427): “The purity, was determined by flow cytometry and ranged between 85% and 90%.”

Statistical comparisons are not properly presented. In particular, it is not recommended anymore to use stars (**) but putting exact p values, the same for "ns" -> put exact value. Also, if possible, indicating fold change is highly recommended as showing effect size is as important as showing p values.

Thank you feedback on the statistical presentation. In the revised manuscript, we have shown exact p-values alongside asterisks to enhance clarity, as recommended.

Given variability in some data (indicated by measurements in individual mice) - e.g., Fig 5A, how much does a gene deletion must impact Tfh (or something else) differentiation, so differences become statistically significant? Seems that with such variability these experiments may be simply underpowered to detect a difference. Experiments in which measurements are "paired" may be thus more powerful (see a suggestion above). This may need to be discussed.

We appreciate the reviewer’s insights and recognize the concern regarding variability in some of our data, particularly in Fig. 5A. We agree that this variability may affect the statistical power needed to detect significant differences in Tfh differentiation and other outcomes. As noted, a paired design with co-transfer of WT and KO cells into the same recipient mouse would indeed offer greater statistical power by reducing inter-mouse variability and enabling direct comparisons within a shared environment. However, as mentioned earlier, our current setup does not allow for co-transfer of WT and KO cells in the same mouse due to the lack of distinct congenic markers. While our findings still offer valuable insights, we acknowledge that future studies with paired designs using distinct congenic markers could improve statistical robustness and clarity.

**Reviewer #2:**

This study carefully evaluates the potential role of Maf1, Myo1f and Prr13 on CD4 T cell maturation following Plasmodium chabaudi chabaudi AS (PcAS) infections in mice. All three genes had previously been shown to be upregulated during T cell differentiation in response to PcAS. They assessed both Plasmodium specific-TCR transgenic and polyclonal T cells and found the anticipated decrease in Tfh cell production following Maf1 deletion in Transgenic CD4+ cells, but neither Myo1f nor Prr13 deletion had a measurable effect. Myo1f or Prr13 have not been tested before for a role in T cell activity so this well-designed study advances the understanding of genes that although upregulated are not critical for CD4 T cell differentiation in response to malaria.

We appreciate the reviewer’s summary and positive assessment of our study. We are pleased that the reviewer recognized the novelty of examining Myo1f and Prr13 in T cell biology, as well as the careful design and contribution of our findings to the understanding of CD4^+^ T cell differentiation in malaria.
