# Supplementary material for: Myosin 1f and Proline-rich 13 are transcriptionally upregulated yet functionally redundant in CD4+ T cells during blood-stage Plasmodium infection
Source: PLoS One. 2025 Mar 25;20(3):e0320375. doi: 10.1371/journal.pone.0320375 (PMC11936294; doi:10.1371/journal.pone.0320375)
Supplement: Attachment — Submitted filename: Rebuttal letter - 2nd revision.docx [file pone.0320375.s004.docx]

Rebuttal letter - 2^nd^ revision

PONE-D-24-38309R1

Myosin 1f and Proline-rich 13 are transcriptionally upregulated yet functionally redundant in CD4+ T cells during blood-stage Plasmodium infection.

We thank the editor and reviewers for their comments and suggestions on our manuscript. We have thoroughly addressed all concerns. Below, we provide a point-by-point response to the reviewers’ comments, with our responses highlighted in BLUE text. Where appropriate, we have included new text that have been incorporated into the revised manuscript, along with line numbers for ease of reference.

Journal Requirements:

Please review your reference list to ensure that it is complete and correct. If you have cited papers that have been retracted, please include the rationale for doing so in the manuscript text, or remove these references and replace them with relevant current references. Any changes to the reference list should be mentioned in the rebuttal letter that accompanies your revised manuscript. If you need to cite a retracted article, indicate the article’s retracted status in the References list and also include a citation and full reference for the retraction notice.

Thank you for the reminder. We have reviewed the reference list to ensure its accuracy.

[Note: HTML markup is below. Please do not edit.]

Reviewers' comments:

Reviewer's Responses to Questions

**Comments to the Author**

1. If the authors have adequately addressed your comments raised in a previous round of review and you feel that this manuscript is now acceptable for publication, you may indicate that here to bypass the “Comments to the Author” section, enter your conflict of interest statement in the “Confidential to Editor” section, and submit your "Accept" recommendation.

Reviewer #1: (No Response)

Reviewer #2: All comments have been addressed

2. Is the manuscript technically sound, and do the data support the conclusions?

The manuscript must describe a technically sound piece of scientific research with data that supports the conclusions. Experiments must have been conducted rigorously, with appropriate controls, replication, and sample sizes. The conclusions must be drawn appropriately based on the data presented.

Reviewer #1: Partly

Reviewer #2: Yes

3. Has the statistical analysis been performed appropriately and rigorously?

Reviewer #1: I Don't Know

Reviewer #2: Yes

4. Have the authors made all data underlying the findings in their manuscript fully available?

The [PLOS Data policy](http://track.editorialmanager.com/CL0/http:%2F%2Fwww.plosone.org%2Fstatic%2Fpolicies.action%23sharing/1/010f0194762830a8-a3812a7e-f0b0-4ec1-af15-9438756b0fe4-000000/7Mt1a7madlpBV_qGzQfas1lAlDQ3M5OjdW1-ejzaPkY=194) requires authors to make all data underlying the findings described in their manuscript fully available without restriction, with rare exception (please refer to the Data Availability Statement in the manuscript PDF file). The data should be provided as part of the manuscript or its supporting information, or deposited to a public repository. For example, in addition to summary statistics, the data points behind means, medians and variance measures should be available. If there are restrictions on publicly sharing data—e.g. participant privacy or use of data from a third party—those must be specified.

Reviewer #1: No

Reviewer #2: Yes

5. Is the manuscript presented in an intelligible fashion and written in standard English?

PLOS ONE does not copyedit accepted manuscripts, so the language in submitted articles must be clear, correct, and unambiguous. Any typographical or grammatical errors should be corrected at revision, so please note any specific errors here.

Reviewer #1: Yes

Reviewer #2: Yes

6. Review Comments to the Author

Please use the space provided to explain your answers to the questions above. You may also include additional comments for the author, including concerns about dual publication, research ethics, or publication ethics. (Please upload your review as an attachment if it exceeds 20,000 characters)

Reviewer #1: Comments.

1. The authors provided some justification of why these 2 genes (Myo1f and Prr13) were perhaps selected for further analysis but their abstract and most of text still did not explicitly say that prior knowledge was used. In particular, abstract ignores this completely. Please revised and put explicit statements that selection of these 2 genes was also driven by prior knowledge of importance of these genes in T cell responses and not just by scRNAseq data (which give hundreds of unregulated genes).

We have now revised the Abstract and relevant sections to explicitly state that selection of *Myo1f* and *Prr13* was informed by both their strong upregulation in the scRNA-seq data, and prior knowledge of their roles in eukaryotic cell biology. Please see the updated abstract (Lines 19-21) and manuscript sections at line 216, line 270-272, and line 320-322.

2. I understand your points of choosing day 7 to measure responses but I think you do not state that clearly in the main text. Please do.

Thank you for pointing this out. To address your comment, we have revised the main text (line 143-145) to clearly explain the rationale for selecting Day 7 post-infection as the critical timepoint for assessing CD4^+^ T cell differentiation.

3. The study could be underpowered - as authors agreed, so this should be listed in the Discussion, along with the point that you could not do experiments when co-transferring WT and KO cells due to cogenic marker identity.

Thank you for your comment. We acknowledge that our study may be underpowered and have now explicitly stated this limitation (line 332-336) in the Discussion.

4. I appreciate showing original gels but I still could not understand them. It could be my limitation, so editor may need to show these to the right expert to ensure they are accurate.

Thank you for your feedback. We clarified our gel data in the previous revision by providing the original gels (in supplemental information) with minimal cropping and adjustments (Figure 3C). We leave it to the Editor’s discretion to request further evaluation if needed.

To clarify again, the distinction between WT and KO groups is based on Cre expression, as both groups carry floxed alleles (f/f) for Myo1f or Prr13. In this study and figures, Myo1f WT (Myo1f(f/f)) lacks Cre expression, while Myo1f KO (CD4-Cre Myo1f(f/f)) expresses Cre. Similarly, Prr13 WT is shown as Prr13(f/f), and Prr13 KO as CD4-Cre Prr13(f/f).

Minor comments

You did not format the figures/captions properly as we do in grants (perhaps you don't apply for funding). You MUST show the figure and caption together! (And preferably on the page near where that figure is first cited). I will not review another paper that is not formatted to help easier review process.

Thank you for your feedback. We apologise for any inconvenience. We have now formatted the figures and their captions together, and positioned them closer to their first mention in the text.

If Figure 1 has been published before (per disclaimer listed), why do we need it here? The authors anyway state that they focused on the genes found by mining previously published dataset (along with some prior knowledge), so do we need to see Figure 1? NOTE: not all panels in Figure 1 are labeled, e.g., Violin plots in A are not. And, you may want to mention that only about 50% of cells (based on shape of violin plots) express Myo1f (the middle panel in Figure 1A) -> is that important that 50% of cells do not upregulate this gene?

Thank you for your comments. While a version of the schematic in Fig 1A has been published, we believe its inclusion is very helpful for readers to easily interpret the transcriptional information in the figure. We would prefer to retain this panel.

To improve clarity, we have now ensured that all panels in Figure 1, including the violin plots in Figure 1A, are properly labelled.

Regarding Myo1f expression, the Reviewer is absolutely correct in pointing out that a proportion of PbTII cells do not express this gene at day 7. This highlighted to us that *Myo1f* expression was largely restricted to Th1 cells, particularly when compared to *Prr13.* The specific pattern of expression encouraged us to select *Myo1f* for functional testing. Text highlighting Th1-restriction can be seen at Lines 101-102.

Please remove "ns" from all panels and put actual p values there. You did not interpret my comment correctly - do not use shortcuts but put actual p values, i.e., p=0.89 if difference is statistically non-significant.

Thank you for the clarification. We have now removed "ns" from all panels and replaced it with the actual p-values, as requested.

The experiments with co-transfer of WT and KO cells could still perhaps be done (in the future) by using some type of cell label, e.g., CTV. The label does get diluted but CD4s are not expected to divide that much, so some level of labeling may be retained by day 7. Perhaps it is worth mentioning as future directions in the paper.

Thank you for your suggestion. While cell-tracing dyes such as CTV can provide short-term labelling, their reliability for distinguishing WT and KO PbTII cells by Day 7 post-infection is uncertain due to progressive dilution from cell proliferation. Day 7 post-infection is the ideal timepoint for us to assess PbTII differentiation because it represents peak cell expansion and the stage when Th1 and Tfh subsets are most distinct. PbTII cells undergo multiple rounds of division upon activation, leading to significant CTV dilution. By Day 7, the CTV signal in hours hands is too weak to reliably distinguish WT from KO cells.

Instead, we have discussed in the Discussion section (line 335) that future studies could use distinct congenic markers to enable reliable co-transfer experiments. This approach would allow direct comparisons within the same recipient mouse without the limitations of dye dilution, ensuring robust and reproducible analyses of PbTII differentiation.

Line 113: do you mean "our data" or "our analysis"? Data had been published.

Thank you for your comment. We intended to refer to our analysis. To clarify this, we have revised the text to explicitly state 'our analysis' instead of 'our data' in line 115 (previously line 113).

Line 139: "infected with PcAS infection" sounds weird. Buttery butter.

We have now revised in line 141 (previously line 139).

Line 145: you must include justification of why day 7 is a good day to measure this response.

We have now explicitly justified the selection of Day 7 in the revised text in line 143-145.

Line 311: include justification of why these genes are good ones by citing relevant other studies.

Thank you for your suggestion. We have now included additional justification for selecting Myo1f and Prr13 by citing relevant studies in line 319-321 (previously line 311).

340: "regulated" -> unregulated (?)

Thank you for your comment. In this context (line 354 in the revised version), we have removed “mRNA is highly regulated”, and replaced with the phrase “mRNA species are variously subjected to post-transcriptional regulation”, which better captures the complex biology surrounding conversion of mRNA to protein.

Reviewer #2: The rationale and figures have been clarified and the authors have responded to all the criticisms within the limits of the genetics of the experimental design.

We appreciate your positive feedback.

7. PLOS authors have the option to publish the peer review history of their article ([what does this mean?](https://track.editorialmanager.com/CL0/https:%2F%2Fjournals.plos.org%2Fplosone%2Fs%2Feditorial-and-peer-review-process%23loc-peer-review-history/1/010f0194762830a8-a3812a7e-f0b0-4ec1-af15-9438756b0fe4-000000/dtxTFlMbmPv_tLzJMZylgqHMveq8sCOw2fofrOaLq9c=194)). If published, this will include your full peer review and any attached files.


If you choose “no”, your identity will remain anonymous but your review may still be made public.


**Do you want your identity to be public for this peer review?** For information about this choice, including consent withdrawal, please see our [Privacy Policy](https://track.editorialmanager.com/CL0/https:%2F%2Fwww.plos.org%2Fprivacy-policy/1/010f0194762830a8-a3812a7e-f0b0-4ec1-af15-9438756b0fe4-000000/UdZjoPaY8wcVmZVazspErSHNADO4xxEGhl7juUB6Nf0=194).

Reviewer #1: No

Reviewer #2: No
